# Measuring neutron capture cross sections of radioactive nuclei: From activations at the FZK Van de Graaff to direct neutron captures in inverse kinematics with a storage ring at TRIUMF

**DOI:** 10.1140/epja/s10050-023-01012-9

**Published:** 2023-05-12

**Authors:** Iris Dillmann, Oliver Kester, Richard Baartman, Alan Chen, Tobias Junginger, Falk Herwig, Dobrin Kaltchev, Annika Lennarz, Thomas Planche, Chris Ruiz, Nicole Vassh

**Affiliations:** 1 TRIUMF, Vancouver, BC V6T 2A3 Canada; 2 Department of Physics and Astronomy, University of Victoria, Victoria, BC V8P 5C2 Canada; 3 Department of Physics and Astronomy, McMaster University, Hamilton, ON L8S 4M1 Canada

## Abstract

Measuring neutron capture cross sections of radioactive nuclei is a crucial step towards a better understanding of the origin of the elements heavier than iron. For decades, the precise measurement of direct neutron capture cross sections in the “stellar” energy range (eV up to a few MeV) was limited to stable and longer-lived nuclei that could be provided as physical samples and then irradiated with neutrons. New experimental methods are now being developed to extend these direct measurements towards shorter-lived radioactive nuclei ($t_{1/2}<$ 1 y). One project in this direction is a low-energy heavy-ion storage ring coupled to the ISAC facility at TRIUMF, Canada’s accelerator laboratory in Vancouver BC, which has a compact neutron source in the ring matrix. Such a pioneering facility could be built within the next 10 years and store a wide range of radioactive ions provided directly from the existing ISOL facility, allowing for the first time to carry out direct neutron capture measurements on short-lived isotopes in inverse kinematics.

## Introduction

The ultimate goal of all world-wide efforts in Nuclear Astrophysics is a complete understanding of the astrophysical origin and the production processes that are responsible for the creation of all the visible matter around us. This quest is also strongly connected to the theoretical understanding of the quantum many-body problem of the atomic nucleus that would enable a reliable prediction of the properties of all nuclei from the proton- to the neutron-dripline.

The interpretation of the observed solar abundances from hydrogen up to uranium is a long-standing problem. The basic foundation was already laid in 1957 by the seminal papers of Cameron [1] and Burbidge et al. [2]. Since then, generations of stellar modellers have worked hand in hand with experimental nuclear astrophysicists and theoretical nuclear physicists to better understand the underlying astrophysical processes and to finetune and constrain the astrophysical and nuclear physics inputs.

Heavy nuclei beyond iron are produced by different reactions mechanisms in different astrophysical scenarios. The main processes driving the nucleosynthesis in these mass regions are neutron capture processes. They are distinguished by the timescale of the capture relative to $\beta$-decay, as well as the neutron densities.

In the “slow” neutron capture (*s*) process ($N_n\approx$ $10^6-10^{12}$ $\hbox {cm}^{-3}$) the neutron capture timescale is slow (in the order of years) compared to the $\beta$-decay half-life, thus the reaction path runs close to the line of stability [3]. In contrast to that, the reaction flow of the “rapid” neutron capture (*r*) process ($N_n>>10^{20}$ $\hbox {cm}^{-3}$) proceeds far off stability through short-lived, neutron-rich nuclei on a much faster neutron capture timescale (ms) than the $\beta$-decay rates [4].

**Fig. 1:**
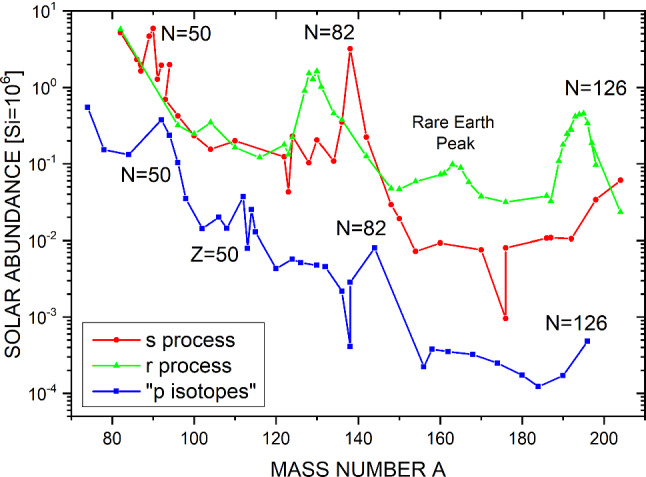
Isotopic solar abundances of heavy elements (taken from Ref. [8]), divided up into the three main processes. The abundance peaks are connected to neutron shell closures

The *s* and the *r*-processes are responsible for production of the majority ($\approx 99$%) of the observed solar abundances heavier than iron (see Fig. 1). For the remaining $\approx 1\%$, various processes on the neutron-deficient side are summarized under the term “*p* processes” [5], among them photo-dissociation reactions ($\gamma$ process), neutrino-induced processes ($\nu \hbox {p}$ and $\nu$ process), as well as rapid proton captures (*rp* process).

Recently, a third neutron capture process has been (re-)discovered and gained a lot of attention. It can nicely explain the abundance pattern in certain carbon-enhanced metal-poor stars which show an enhancement in both, *r*-process (Eu) and *s*-process (Ba) elements (CEMP-r/s stars). This so-called “intermediate” neutron capture (*i*) process ($N_n\approx$ $10^{13}-10^{16}$ $\hbox {cm}^{-3}$) lies in-between the *s*- and the *r*-process reaction paths, just a few mass units away from stability [6, 7].

In the following Sect. 2 a short overview will be given about key nuclei for a better understanding of the respective neutron capture processes, as well as key challenges for the measurement of these cross sections. Section 3 will try to summarize the status and present knowledge of neutron capture cross sections, while Sect. 4 will introduce a potential pioneering facility at TRIUMF to measure neutron capture cross sections of short-lived nuclei in a storage ring.

## Neutron capture cross sections in nuclear astrophysics

The most important nuclear physics input for all three neutron capture processes (*s*, *r*, and *i*) are neutron capture cross sections in the stellar energy range (keV to a few MeV) as well as decay half-lives. The solar abundance curves for the *s*-, *r*-, and *p*-process isotopes in Fig. 1 show the characteristic abundance peaks at the neutron-shell closures $N=50$, 82, and 126. This connection between an astrophysical observable (the measured abundances) and a nuclear structure phenomenon (shell closures) has already been identified in the 1950s in the seminal B$^2$FH review [2].

Due to the decreasing neutron binding energy towards the neutron dripline the respective neutron capture cross section steadily decreases. However, directly at the neutron shell closures it shows a steeper drop due to the increased binding energy. This localized smaller neutron capture cross section acts as bottleneck for the reaction flow and allows accumulation of material at these neutron-magic isotopes, as evidenced by the abundance peaks at $A=90$, 138, 208 for the *s*-process and $A\approx 80$, 130, and 195 for the *r*-process.

Whereas for the *s*-process reaction flow only nuclei along the line of stability contribute (which are largely known experimentally—see Sect. 2.2), the *i*- and *r*-process calculations require a knowledge of cross sections of short-lived neutron-rich nuclei which—to a large extent—have not yet been measured.

### Neutron captures in stellar conditions

In an astrophysical environment with stellar temperature *T*, however, all particles are thermalized due to interactions with the photon bath and their energy distribution can be described with a Maxwell-Boltzmann distribution.

Accordingly, the astrophysical neutron capture cross section averaged over this energy distribution are called “Maxwellian-averaged cross sections” (MACS) and have been tabulated for *s*-process temperatures between $kT= 5-100$ keV, e.g. in Ref. [9] or in the https://exp-astro.physik.uni-frankfurt.de/kadonis1.0/KADoNiS database (Karlsruhe Astrophysical Database of Nucleosynthesis in Stars).

It should be emphasized that the vast majority of the directly measured laboratory cross sections so far have been done only with the target nucleus in the ground-state (exception: quasi-stable $^{180m}\hbox {Ta}$). However, the high-energy tail of the Maxwell-Boltzmann distribution allows also thermal population of low-lying excited states which can strongly impact the respective stellar neutron capture cross section. Earlier works have tried to describe the translation into a stellar cross section with help of a (temperature-dependent) “stellar enhancement factor” (SEF) [9]. This factor has been calculated by comparison of the MACS of the nucleus in the ground state with the the respective nucleus in a thermal equilibrium.

**Fig. 2:**
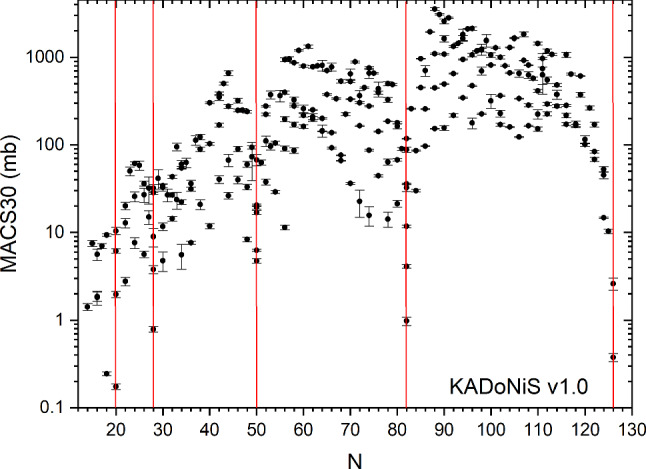
Experimentally measured Maxwellian-averaged cross sections at $kT= 30$ keV (MACS30) for all nuclei between $^{28}\hbox {Si}$ and $^{209}\hbox {Bi}$. Data taken from the https://exp-astro.physik.uni-frankfurt.de/kadonis1.0/KADoNiS database

To allow a better guidance how useful the measurement of the neutron capture cross section of a nucleus in the ground-state is to constrain the stellar rate, the “ground-state contribution factor” $X_i$ was later introduced [10]. Its values are tabulated (along with the SEF) in the https://exp-astro.physik.uni-frankfurt.de/kadonis1.0/KADoNiS database. If the factor $X_i$ is close to unity, the ground-state rate dominates the stellar rate at the given temperature. Figures 2 and 3 in Ref. [10] show the factor for 380 MK (*s*- and *i*-process temperatures) and for 2.5 GK (*r*- and $\gamma$-process temperatures). Especially deformed mid-shell nuclei like the lanthanides in the mass region *A* = 150–180 have low-lying excited states that are easily populated and thus lower the importance of the contribution of the ground-state to the stellar rate.

For a more detailed description about *“Stellar neutron capture reaction at low and high temperatures”* the reader is referred to a recent publication in this journal [11].

### Present experimental status

Neutron capture cross sections need to be measured in the stellar energy range, ranging from a few hundred eV to $\approx$2 MeV for the *s* and the *i* process. Due to its location close to stability, the majority of the required cross sections for the *s*-process have been measured in the past decades in or close to the astrophysically relevant energy range. An overview about all measured MACS at $kT= 30$ keV (MACS30 values) for 265 nuclei between $^{28}\hbox {Si}$ and $^{209}\hbox {Bi}$ is shown in Fig. 2.

**Fig. 3:**
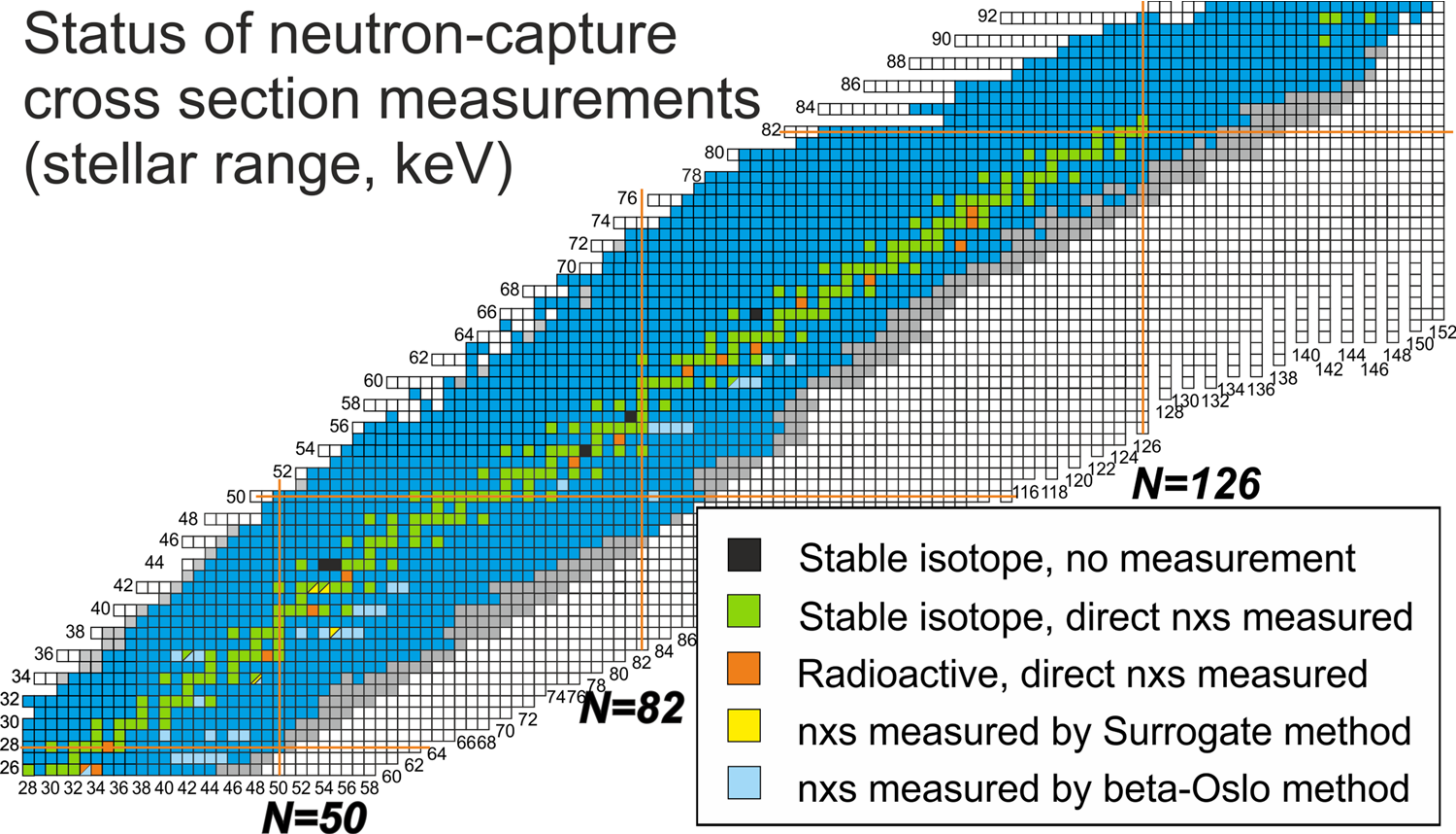
Chart of nuclides with status of half-life measurements (blue boxes) as well as newly identified nuclei without any measurement (grey boxes). Various types of direct and indirect neutron capture cross section measurements are indicated by different colors

For stable nuclei heavier than iron almost all neutron capture cross sections at *s*-process temperatures have been determined, with the exception of $^{98,99}\hbox {Ru}$, $^{131}\hbox {Xe}$, $^{138}\hbox {La}$, and $^{158}\hbox {Dy}$ (black boxes in Fig. 3). Overall, so far only 16 long-lived radioactive neutron capture cross sections have been measured with direct methods for astrophysical purposes between $Z=26-82$ (orange boxes in Fig. 3).

Important for a direct measurement is the ability to create a “physical” sample with enough nuclei of interest. For stable isotopes this does not pose a problem since the material can be commercially acquired as elemental sample with natural abundances or as enriched sample material. Samples of long-lived nuclei, however, need to be produced first (in the majority of cases via neutron-induced reactions in reactors), and then purified via chemical (isobaric) separation before they can be used again for the respective neutron capture measurement. Depending on the half-life of the isotope of interest, the chemical separation step needs to be repeated if the time interval between the production of the sample and the irradiation is “too long” and decay daughters have been produced that would interfere with the planned measurement (e.g. due to their high activity or unfavorable large cross sections or $\gamma$-lines in the region of interest). Even with purified (mono-isotopic) samples the high intrinsic activity of the short-lived radioactive nuclei can still hamper the direct measurement.

Recent examples for measurements on short-lived radionuclides are

- $^{63}\hbox {Ni}(n,\gamma )^{64}\hbox {Ni}$ ($t_{1/2}= 101.2$ y), measured at CERN n_TOF with a sample of 112 mg, corresponding to $1.1\times 10^{21}$ atoms $^{63}\hbox {Ni}$ (activity 240 GBq) [12].
- $^{171}\hbox {Tm}(n,\gamma )^{172}\hbox {Tm}$ ($t_{1/2}= 1.92$ y), also measured at CERN n_TOF with a sample of 3.1 mg, corresponding to $10^{19}$ atoms with an activity of 114 GBq [13].
- $^{60}\hbox {Fe}(n,\gamma )^{61}\hbox {Fe}$ ($t_{1/2}= 2.62$ My), measured at FZK Karlsruhe with the activation method with a sample of 1.35 $\mu \hbox {g}$, corresponding to $1.35\times 10^{16}$ atoms with an activity of 113 Bq [14].
- $^{79}\hbox {Se}(n,\gamma )^{80}\hbox {Se}$ ($t_{1/2}= 326$ ky), also measured by the CERN n_TOF collaboration with a sample size of 3 mg ($2.3\times 10^{19}$ atoms), corresponding to an activity of 1.54 MBq [15].

The $^{179}\hbox {Ta}(n,\gamma )$ cross section ($t_{1/2}= 1.82$ y), however, could only be measured with thermal neutrons using a sample of 60 ng ($2\times 10^{14}$ atoms, [16]).

From this list one can already see that for the direct measurement of neutron capture cross sections in general a minimum sample size of $\approx 10^{15}$ atoms is required. For an isotope with a half-life $t_{1/2}=1$ y this would correspond to an activity of 22 MBq which is still relatively easy to handle. But for shorter-lived nuclei, down to hours or even seconds of half-life, the intrinsic sample activity becomes a major issue for the measurement.

### Comparison to theoretical predictions

Figure 3 shows the present dilemma: decay half-lives are measured for almost all known isotopes (except for the most neutron-rich nuclei that have only recently been identified, shown as grey boxes), but neutron capture cross sections at stellar energies have only been measured for stable and long-lived nuclei along the line of stability.

Recent indirect methods like surrogate methods [17, 18] (yellow boxes) and the $\beta$-Oslo method [19–22] (light blue boxes) have allowed for extending this range to more neutron-rich nuclei but so far only covered a few radioactive nuclei.

**Fig. 4:**
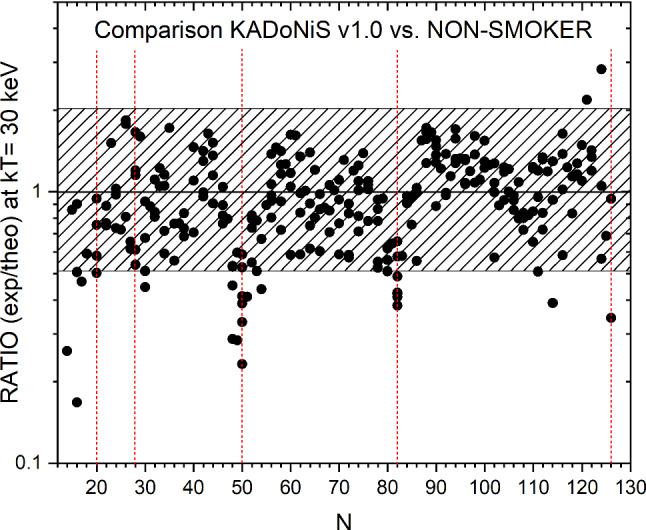
Ratio between experimentally measured MACS30 and the NON-SMOKER predictions for all nuclei between $^{28}\hbox {Si}$ and $^{209}\hbox {Bi}$

How well do theoretical Hauser-Feshbach models [23], like the statistical model code NON-SMOKER [24] reproduce these measured neutron capture cross sections at *s*-process temperatures? In Fig. 4 a comparison of measured MACS30 values of nuclei between $^{28}\hbox {Si}$ and $^{209}\hbox {Bi}$ is given as ratio between the measured value and the NON-SMOKER prediction.

NON-SMOKER can reproduce these experimentally measured cross sections at $kT= 30$ keV within a factor of 2 or better. From the 265 cross sections, only 24 (9.1%) are outside of this window, and the majority of these values are overpredicting the experimental result, especially at the $N=50$ and 82 shell closures. This deficiency of the statistical model is well known and originates from the low level density in these regions around the neutron shell closures which can be solved by microscopic model corrections.

But how does the statistical model perform for neutron capture cross sections outside of the known region? Fig. 2 in Ref. [20] illustrates this for neutron-rich nuclei with another statistical model code (TALYS [25]). The two main ingredients for neutron capture cross sections, nuclear level densities and $\gamma$-strength functions, have been varied within their model parameters. This resulted in ratios between the lowest and the highest predicted neutron capture rate at $T= 1.5$ GK of $>100$ in *i*-process regions just a few mass units away from the well-constrained areas at stability.

These large deviations show the necessity for either measurements of neutron capture cross sections or a better constraint of the respective nuclear ingredients (nuclear level densities and $\gamma$-strength functions) of these shorter-lived isotopes away from the valley of stability.

### Neutron captures in the *s* process

The *s* process follows a well-defined reaction path along the valley of stability up to $^{209}$Bi since neutron captures and subsequent $\beta$-decays occur on a “slow” timescale, [3, 26]. It can be divided into a *weak* component mainly responsible for the creation of nuclei with $A<90$ and a *main* component for heavier nuclei up into the Pb–Bi region. The astrophysical conditions of these two components are vastly different and lead to different neutron densities around $N_n\approx 10^6-10^{12}$ $\hbox {cm}^{-3}$.

The neutrons for the *s*-process nucleosynthesis originate from $\alpha$-induced reactions that are operational under these astrophysical conditions: the $^{22}\hbox {Ne}(\alpha ,n)^{25}\hbox {Mg}$ reaction during core helium and shell carbon burning in the weak *s*-process in massive stars ($M< 8~M_\odot$), and the $^{13}\hbox {C}(\alpha ,n)^{16}\hbox {O}$ reaction (during shell hydrogen burning) and then the $^{22}\hbox {Ne}(\alpha ,n)^{25}\hbox {Mg}$ reaction again (during helium shell flashes) in thermally-pulsing AGB stars for the main *s*-process.

In the *s*-process nucleosynthesis several types of key nuclei can be distinguished which have different impact on the nucleosynthesis process. An accurate knowledge of their cross sections is indispensable for a better understanding of the neutron economy in the *s* process and the resulting calculated abundances.

#### *s*-Only isotopes

Isotopes which are solely produced by the *s* process and not by any other heavy element nucleosynthesis process are called “*s*-only” isotopes. Table 1 in Ref. [27] lists 33 isotopes which are blocked by stable isobars from the respective *r*-process decay chains.

**Fig. 5:**
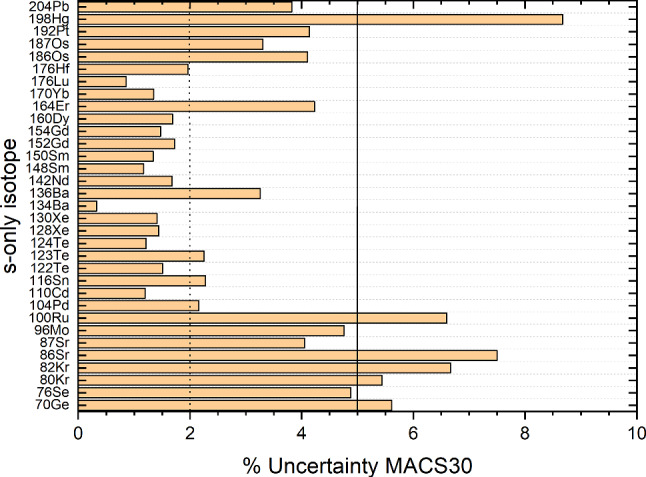
Measured uncertainties (in %) for the MACS30 of the identified *s*-only isotopes

Figure 5 shows the measured uncertainties (in %) for their MACS30 values. Overall, all 33 *s*-only isotopes have been measured with an uncertainty of better than 10%, the mass region $A=100$–180 even reaches 2% or better. The remaining cases which should be measured to a better precision ($<5\%$) are $^{70}\hbox {Ge}$, $^{80,82}\hbox {Kr}$, $^{86,87}\hbox {Sr}$, $^{100}\hbox {Ru}$, and $^{198}\hbox {Hg}$.

#### Nuclei at neutron shell closures

As discussed before, stable nuclei at the neutron shell closures ($N= 50$, 82, 126) exhibit a local minimum in their neutron capture cross section which leads to a bottleneck in the reaction flow and thus accumulation of material. The neutron capture cross sections should be measured with very good precision since they directly impact the width and height of the abundance peak at these mass numbers. The nuclei which are responsible for the *s*-process abundance peaks (see Fig. 1) are $^{86}\hbox {Kr}$, $^{88}\hbox {Sr}$, $^{89}\hbox {Y}$, and $^{90}\hbox {Zr}$ at the $N=50$ shell closure; $^{136,138}\hbox {Ba}$ and $^{140}\hbox {Ce}$ at the $N=82$ shell closure, and $^{208}\hbox {Pb}$ at the $N=126$ shell closure.

**Fig. 6:**
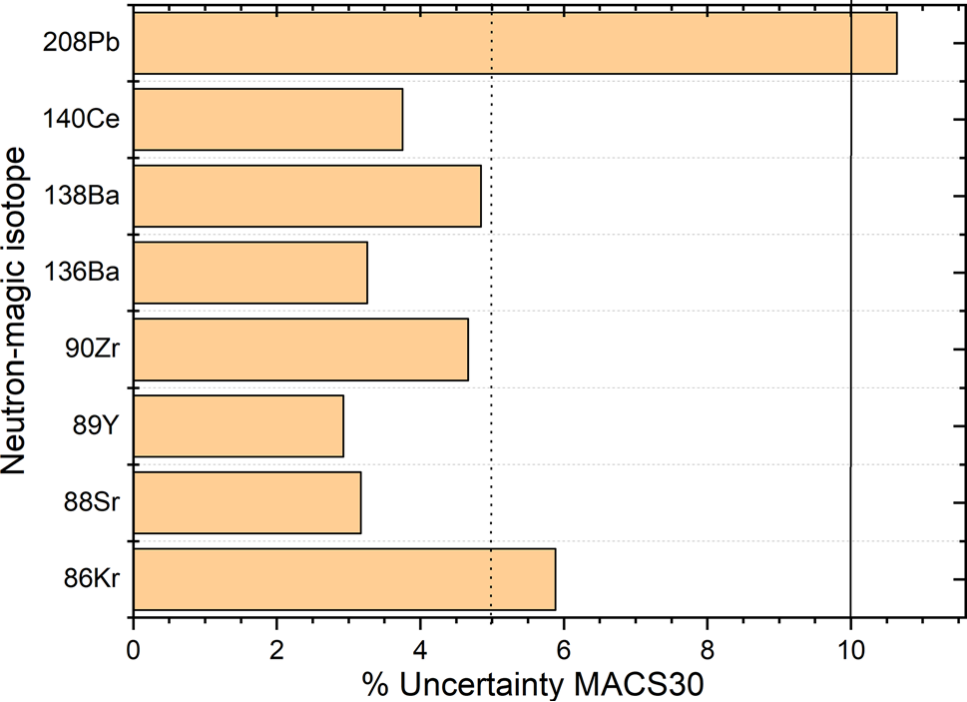
Measured uncertainties (in %) for the MACS30 of the stable isotopes at the $N=50$, 82, and 126 shell closures which are responsible for the peak in the *s*-process abundance curve

All of these cross sections have been measured at $kT= 30$ keV with 3–6% uncertainty, with the exception of $^{208}\hbox {Pb}(n,\gamma )^{209}\hbox {Pb}$ which is only known to 10.5% (see Fig. 6).

#### Bottlenecks: the $^{62}\hbox {Ni}$ case

Also other nuclei with small cross sections can create a bottleneck in the reaction flow. The impact of this propagation effect has been discussed in detail for the weak *s*-process in Ref. [28] and shown e.g. for the $^{62}\hbox {Ni}(n,\gamma )^{63}\hbox {Ni}$ cross section in Fig. 2 of Ref. [29]. An earlier measurement based on a private communication lead to a recommended MACS30 of $12.5\pm 4.0$ mb which —when used in weak *s*-process calculations—acted as a bottleneck and strongly hindered the reaction flow towards heavier masses up to mass $A=90$. This “stellar $^{62}\hbox {Ni}$ problem” could be solved by subsequent cross section measurements with different methods, e.g. in Refs. [29, 30] using neutron activations followed by measurement of the long-lived activation product $^{63}$Ni with Accelerator Mass Spectrometry, or via the time-of-flight method in Refs. [31, 32].

These four independent measurements showed that the $^{62}\hbox {Ni}(n,\gamma )^{63}\hbox {Ni}$ cross section is about a factor of two larger than previously thought, leading to the now recommended value of $\hbox {MACS30}= 22.2\pm 1.3$ mb from Ref. [32] in the KADoNiS database. This higher cross section reduces the bottleneck at $^{62}\hbox {Ni}$ and allows about 30% more *s*-process material to flow to higher masses up to $A=90$ compared to the previously used lower cross section, as shown in Fig. 3 in the paper of Nassar et al. [29].

#### Neutron absorbers and neutron poisons

While nuclei with small neutron capture cross sections hinder the reaction flow towards heavier masses, nuclei which are either produced in large quantities and/or have very large cross sections also play a pivotal role as “neutron absorbers” or “neutron poisons” and impact the neutron economy of the *s* process.

The difference between these two types of neutron-consuming nuclei is if they bind the neutrons with or without bringing them back into the reaction cycle. Generally speaking, neutron poisons are light nuclei that are produced in large quantities and capture neutrons that then are no longer available for capture of heavier *s*-process seed nuclei. Reactions on abundant isotopes like $^{14}\hbox {N}(n,p)^{14}\hbox {C}$ or $^{25}\hbox {Mg}(n,\gamma )^{26}\hbox {Mg}$ take the neutrons out of the *s* process irrevocably.

Neutron absorbers, however, recycle the neutrons in a subsequent reaction step, e.g. $^{12}\hbox {C}(n,\gamma )^{13}\hbox {C}$ and $^{13}\hbox {C}(\alpha ,n)^{16}\hbox {O}$, or $^{16}\hbox {O}(n,\gamma )^{17}\hbox {O}$ [33] and $^{17}\hbox {O}(\alpha ,n)^{20}\hbox {Ne}$. While the neutron capture cross sections on $^{12}\hbox {C}$ and $^{16}\hbox {O}$ are quite small ($14.3\pm 1.1$ $\mu \hbox {b}$ and $34.9\pm 0.7$ $\mu \hbox {b}$, respectively, at $kT=30$ keV), their seed abundances at the beginning of the *s*-process burning phases are relatively high compared to other nuclei. Thanks to the subsequent ($\alpha$,n) reaction the captured neutrons are then cycled back into the *s* process.

The correct treatment of these nuclei in *s*-process calculations thus not only requires precise measurements of the respective neutron capture cross section but also on the subsequent $(\alpha ,n)$ reaction that cycles the neutrons back into the process. Recent measurement for the $^{13}\hbox {C}(\alpha ,n)^{16}\hbox {O}$ reaction can be found in Refs. [34, 35], and for $^{17}\hbox {O}(\alpha ,n)^{20}\hbox {Ne}$ in Refs. [36, 37]

#### Radioactive nuclei

The measurement of neutron capture rates on radioactive nuclei is still the biggest challenge for the *s* process (as well as any other neutron capture process—see following Sects. 2.5 and 2.6). Of special interest among all of these *s*-process key nuclei are of course those radioactive nuclei which create diversions in the reaction path, the so-called “branching-point nuclei”. These nuclei have half-lives comparable to the neutron capture timescale in the *s* process ($\lambda _\beta \approx \lambda _{n,\gamma }$).

**Fig. 7:**
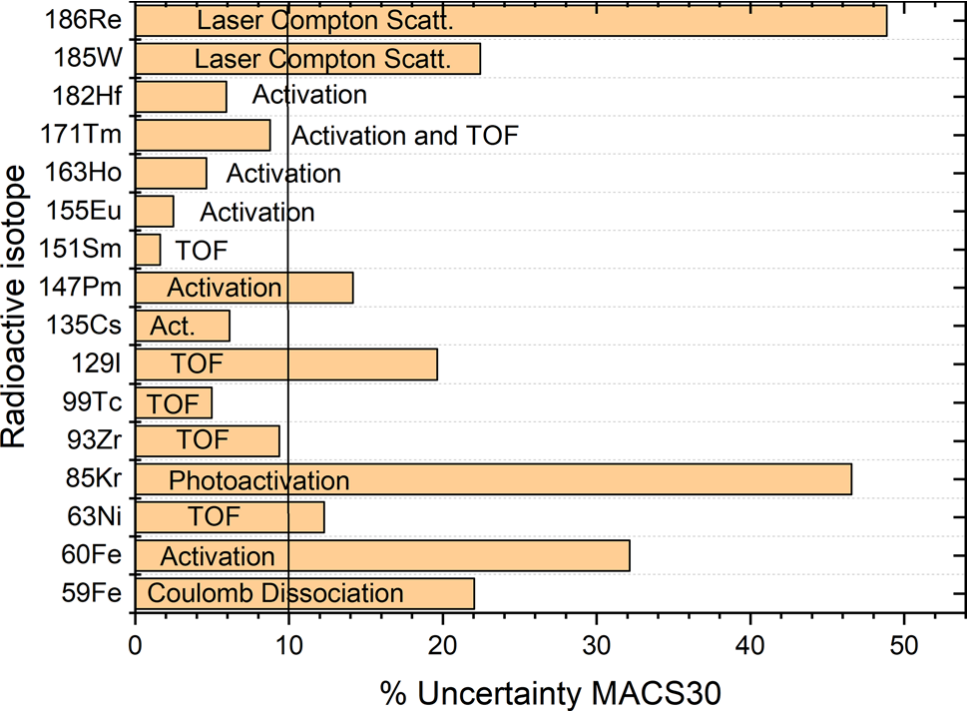
Measured uncertainties (in %) for the MACS30 of radioactive nuclei in the *s*-process path

Direct neutron capture measurements on radioactive nuclei for the *s*-process have been limited so far by the availability of the respective radioactive samples. An overview about the achieved uncertainties (at *kT*= 30 keV) is given in Fig. 7.

Photon-induced $(\gamma ,n)$ reactions have also been used as an alternative to extract $(n,\gamma )$ cross sections for short-lived nuclei, albeit with larger uncertainties, e.g. with laser Compton scattering [38] or Bremsstrahlung photons [39, 40]. For the determination of the $^{59}\hbox {Fe}$ cross section ($t_{1/2}= 44.5$ d) a Coulomb dissociation measurements has been carried out at GSI Darmstadt [41]

### Neutron captures in the *i* process

Recent astrophysical models and observations in CEMP-r/s stars indicate the presence of a third neutron capture process with *“intermediate”* neutron densities of $N_{n}\approx$ $10^{15}$ $\hbox {cm}^{-3}$[42, 43]. The CEMP stars are “fossil” low-metallicity stars that do not yet carry the signatures of many cycles of nucleosynthesis and thus provide a unique insight into the fundamental building blocks of stellar nucleosynthesis.

The *i* process itself was first described in 1977 to occur in He-shell flashes in thermally-pulsing asymptotic giant branch (TP-AGB) and post-AGB stars where the $^{13}\hbox {C}(\alpha ,n)^{16}\hbox {O}$ neutron source is activated [6]. Thermal convection carries hydrogen from the surrounding hydrogen-rich envelope into the helium burning shell and triggers the production of neutrons with the reaction sequence $^{12}\hbox {C}(p,\gamma )^{13}\hbox {N}(e^+ \nu )^{13}\hbox {C}(\alpha ,n)^{16}\hbox {O}$. More recent studies indicate that Rapidly Accreting White Dwarfs (RAWD) in close binary systems are also a possible scenario [44].

The reaction flow of the *i* process proceeds through radioactive nuclei 2–6 neutrons away from stability where the majority of nuclear properties (masses, half-lives) are known experimentally, except for neutron capture cross sections. These have to be inferred from Hauser-Feshbach models (e.g. NON-SMOKER [24] and TALYS [25]).

Recent Monte Carlo abundance calculations of the University of Victoria group have identified several neutron capture reactions which impact the *i*-process abundances. These nuclei either act as bottleneck and hinder the reaction flow to heavier masses, e.g. $^{66}\hbox {Ni}(n,\gamma )$, or show strong correlations with the resulting calculated abundances in CEMP-i stars, e.g. $^{75}\hbox {Ga}(n,\gamma )$ (strong correlation with the As abundance) [45], $^{135}\hbox {I}(n,\gamma )$ and $^{137}\hbox {Cs}(n,\gamma )$ (strong correlation with the Ba abundance), or $^{141}\hbox {Ba}(n,\gamma )$ and $^{141}\hbox {La}(n,\gamma )$ (strong correlation with the Pr abundance) [46].

The Brussels group has also carried out detailed *i*-process simulations for AGB stars and investigated the dependence on the $^{13}\hbox {C}(\alpha ,n)^{16}\hbox {O}$ rate as well as the ingredients of neutron capture rates (photon strength function, level densities, and direct capture) [47–49].

There is an active experimental program underway at various facilities for the indirect measurement of neutron capture rates on the neutron-rich side. Several $\beta$-Oslo measurements have been carried out in the past years (see, e.g. Refs.  [19–22]) as well as surrogate measurements (see, e.g. [17, 18, 50]). Figure 3 shows this recent progress with yellow and light blue boxes.

### Neutron captures in the *r* process

The biggest obstacle of the *r*-process is that its reaction flows proceed through the so-called *“Terra Incognita”* with thousands of nuclei where no experimental information exists yet. However, not all of these nuclei need to be studied for a better understanding of *r*-process nucleosynthesis.

Part of this *“Terra Incognita”* of nuclides will become accessible in the coming years with the operation of the new generation of radioactive beam facilities, and a multitude of measurements of masses, $\beta$-decay half-lives, and $\beta$-delayed neutron emission probabilities on neutron-rich nuclei in or near the *r*-process region will be performed. But even with these new facilities, a direct measurement of neutron capture cross sections of short-lived nuclei will remain a major challenge.

During the *r* process the individual neutron capture cross sections do not play a role as long as the temperature and neutron density are high and the $(n,\gamma )\leftrightarrow (\gamma ,n)$ equilibrium holds (“detailed balance”). During this phase, only the neutron separation energies determine the location of the flow.

Once the *r* process drops out of equilibrium (“Freeze-out”), the remaining neutrons are captured quickly and re-shape the final abundance curve. These recaptured neutrons lead e.g. to a “smoothing” of the previous odd-even staggering in the abundances (see, e.g. Ref. [51]).

Sensitivity studies using Monte Carlo variations for different nuclear physics parameters such as masses, $\beta$-decay half-lives, neutron-capture rates, and $\beta$-delayed neutron emission probabilities have identified key regions for future studies [52]. These regions are located around the $N=50$, 82, and 126 shell closures, and in the lanthanide region which are connected to the observed abundance peaks at $A=80$, 130, and 195, as well as a smaller “rare earth peak” at $A\approx 160$.

The work by Ref. [53] shows the effects of the reaction rate uncertainties on the *r*-process abundance curve. A factor of 10 uncertainty in the neutron captures is already leading to a smear-out effect that obstructs any fine details of the abundance pattern.

## New methods for the measurement of short-lived nuclei

For the measurement of neutron capture rates on radioactive nuclei “inverse kinematics” has to be employed. In “normal kinematics” one would produce a neutron beam and activate a sample with the isotope of interest and measure the decay or reaction products. As mentioned before, for short-lived nuclei one can no longer create a large enough “physical” sample for this method. Inverse kinematics measurements at radioactive beam facilities are one possibility to circumvent this problem.

The largest obstacle so far for direct neutron capture reactions in inverse kinematics is the creation of a suitable neutron “target” to carry out a “direct” neutron capture measurement. Due to this, indirect nuclear reactions have been employed instead where the neutron is replaced by a heavier target atom, e.g. deuterium, or the formation cross section of states in a common compound nucleus is investigated. Nuclear theory is then used to infer the respective $(n,\gamma )$ cross section from the measured reaction rates or nuclear properties.

### Surrogate methods

The surrogate method was first introduced to extract neutron-induced fission (*n*, *f*) cross sections [54]. In the past decade this method has gained great popularity for its application to extract neutron capture cross sections, especially for short-lived nuclei. For a review article about this method, please refer to Escher et al. [17].

The surrogate method uses the fact that the reaction proceeds via an excited compound nucleus which then decays by the emission of particles and $\gamma$ rays. The measured decay probabilities are then combined with calculated formation cross sections for the compound nucleus and the relevant reaction cross section is extracted. Still, the largest uncertainties for neutron capture cross section calculations come from an incomplete knowledge of the underlying nuclear structure parameters of the compound nucleus, namely the $\gamma$-ray strength function and the nuclear level density, which influence the formation cross section and decay modes. These parameters need to be constrained experimentally to make the calculations more reliable.

Early examples of the surrogate method employed the “Weisskopf-Ewing” approximation, which assumes that the decay of the compound nucleus is independent of its spin and parity. However, this method resulted in large disagreements between the extracted and known cross sections, mainly due to differences in the angular momentum (spin and parity) distributions with which compound nuclei are formed in the $(n,\gamma )$ and surrogate reactions.

This spin-parity mismatch is overcome in the study of Ref. [50] where a new description of the (*d*, *p*) reaction channel and its effect on the decay of the compound nucleus is employed. The study used the $^{95}\hbox {Mo}(\hbox {d,p}\gamma$) reaction as a benchmark since it can be compared to the direct $^{95}\hbox {Mo}(n,\gamma )$ cross section which has been measured up to $E_n=200$ keV [55]. The new description achieves excellent agreement with the direct measurement.

The surrogate method is a powerful tool to measure neutron capture cross sections via indirect methods. It is however hampered—like many other reaction-based studies—by the necessity to have relative high beam intensities of $10^{4}$ pps [56].

### Oslo- and $\beta$-Oslo methods

The “Oslo method” is an experimental technique to extract the nuclear level density (NLD) and the $\gamma$-ray strength function ($\gamma \hbox {SF}$) from transfer reactions, e.g. (*d*, *p*). These two quantities are the main sources of uncertainty in the calculation of neutron-capture cross sections within the statistical Hauser-Feshbach model [23]. Without any experimental constraints on these parameters, theoretical predictions for cross sections of neutron-rich nuclei may vary by orders of magnitude.

A similar technique is the “$\beta$-Oslo method” using a radioactive sample implanted into a segmented total absorption spectrometer [19–22]. The detection of the following $\gamma$-rays allows for determination of the total excitation energy of a state populated by the $\beta$-decay. The NLD and $\gamma \hbox {SF}$ can then be inferred from a measurement of the total decay energy of the formed compound nucleus, which in turn can be used to constrain the neutron capture rate.

The technique can be applied to nuclei with production rates down to 10 pps [56] and thus much shorter-lived neutron-rich nuclei can be reached than with the surrogate method.

### Storage ring method

However, one problem that still persists in these “one-pass experiments” is that only a few reactions occur, and the vast majority of the unreacted (and precious) radioisotopes is left unused and dumped.

By injecting the radioactive isotopes into a storage ring this beam could be “recycled” and thus allow the unreacted beam to continue orbiting and repeatedly interact with a (neutron) target. In this way the luminosity can be increased by orders of magnitude (5–6 orders for a ion beam of 100’s of keV to a few MeV) compared to one-pass experiments.

This concept has been described by Reifarth et al. [57, 58] for neutron captures. Analogously to the successful ongoing proton-capture program in inverse kinematics at the Experimental Storage Ring (ESR) at GSI Darmstadt, Germany [59, 60], the radioactive beam from the connected RIB facility is injected into the storage ring where it circulates and repeatedly interacts with the respective neutron target.

So far no facility exists in the world that combines all three sub-facilities (RIB production facility, storage ring, and neutron-production facility). Whereas suitable storage ring facilities exist that are connected to powerful RIB in-flight facilities (e.g. ESR and CRYRING at GSI Darmstadt, CSRe at HIRF in Lanzhou, Rare RI Ring at RIKEN Nishina Center), no storage ring has been installed yet at an ISOL facility. The “TSR at ISOLDE” project [61] has discussed—among other exciting projects —the use of a storage ring for low-energy charged-particle reactions but this exciting project has unfortunately so far not received more support by CERN.

In the 2019 workshop https://indico.cern.ch/event/838820/ “Exploiting the Potential of ISOLDE at CERN” (EPIC) a new storage ring design for ISOLDE has been brought forward by the user community. This project has been submitted as input for the https://europeanstrategyupdate.web.cern.ch/welcomeEuropean Particle Physics Strategy Update 2018–2020, and the next years will show if the European community is willing to support such a unique facility. One possible use of a storage ring at ISOLDE could be in conjunction with the envisioned “Gamma Factory” (GF) at CERN [62]. If the storage ring is installed near the GF and the radioactive beams are injected from the ISOLDE facility, the circulating ion beam and the photon beam can interact collinearly in one of the straight sections. The reaction products are then separated from the circulating beam and detected.

In the conceptual study in Ref. [57] a potential neutron capture ring facility has been discussed in combination with a high-flux reactor where the neutron target is created by the fission neutrons, allowing direct neutron capture measurements down to minutes. The possibility to realize such a facility is however very low, and the proximity to the core of the fission reactor makes the environment very hostile for the measurement of reactions with this method.

A more likely concept has later been described in Ref. [58] using a spallation neutron target that is surrounded by a heavy water ($\hbox {D}_2\hbox {O}$) moderator. The neutrons are produced via spallation reactions on a small tungsten target that is installed perpendicular to the beam pipe with the circulating radioisotopes. In this concept the two beamlines do not intersect since the ion beam pipe is shifted by a few cm off-center. The study varied the moderator and tungsten target sizes and concluded that with a moderator radius of 1 m, a tungsten target of 1.5 cm radius and 10 cm length, and 100 $\mu \hbox {A}$ of the 800 MeV beam at Los Alamos a neutron density of $5\times 10^{9}$ $\hbox {cm}^{-3}$ can be achieved. This number is only a factor of 4 lower than the areal density calculated for the reactor case.

Such a concept is presently discussed at Los Alamos National Laboratory in the USA. For more information, the reader is referred to the recent Los Alamos Laboratory Report by Mosby et al. [63].

## The TRIUMF Storage Ring Project (TRISR)

### The existing ISAC and new ARIEL facility

At TRIUMF, Canada’s particle accelerator centre in Vancouver, the Isotope Separator and ACcelerator (ISAC) facility [64] is the world’s highest power radioactive ion beam facility of the isotope separation on-line (ISOL) type. Rare isotopes are produced by spallation and fragmentation reactions in the ISAC production targets induced by beams of up to 100 $\mu \hbox {A}$ of 500 MeV protons delivered by TRIUMF’s main cyclotron. The reaction products are ionized, mass separated, and delivered to the ISAC-I experimental hall in the form of high-quality, low-energy ($\sim 30$ keV) radioactive ion beams.

These low-energy beams can either be used directly in, for example, high-resolution decay spectroscopy research and high-precision half-life and mass measurements, or accelerated through the ISAC-I radio frequency quadrupole (RFQ) and drift-tube linear (DTL) accelerators to energies of 0.15–1.8 *A* MeV and used in direct measurements of reaction cross sections relevant to nucleosynthesis in explosive stellar environments.

In 2009, the ISAC-II superconducting heavy-ion linear accelerator was completed, increasing the maximum accelerated radioactive ion beam energy to 6 *A* MeV for mass number $A \le 238$ and as high as 15 *A* MeV for light nuclei.

The radioactive beam production facilities at TRIUMF have been undergoing a major expansion over the past decade with the construction of a new high-power (ultimately 35 MeV, 10 mA, 350 kW) superconducting electron linear accelerator (e-linac) and associated infrastructure of the Advanced Rare IsotopE Laboratory (ARIEL) [64]. In 2026 and 2027, the parallel operation of the existing ISAC 500 MeV, 100 $\mu \hbox {A}$ proton beamline, the high-power ARIEL e-linac photofission driver, and a second 500 MeV, 100 $\mu \hbox {A}$ proton beamline to the ARIEL target stations, will establish a unique multi-user capability that will provide first two, and then three simultaneous radioactive beams, ultimately tripling the beamtime available to the suite of state-of-the-art research infrastructure at ISAC.

### The future: A storage ring?

**Fig. 8:**
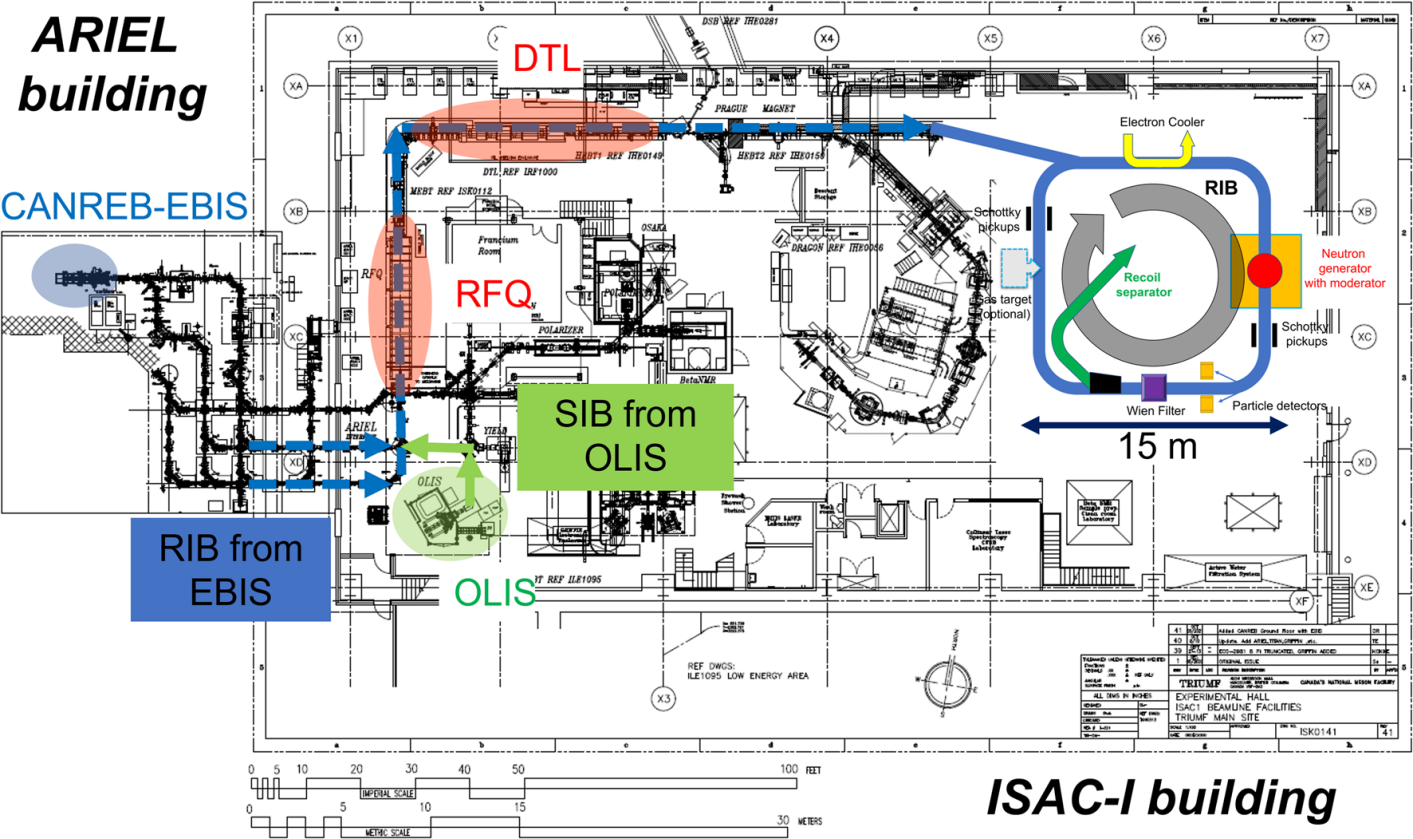
TRIUMF ARIEL and ISAC-I experimental hall with proposed location for the low-energy storage ring (TRISR). The locations of the CANREB-EBIS (in the new ARIEL building), the offline ion source (OLIS), the RFQ, and the Drift Tube Linac (DTL), and potential locations for the main detectors and devices in the ring matrix are shown. The neutron generator is located on the right side (East corner). Stable and radioactive beam paths are partially indicated

The TRIUMF Storage Ring (TRISR) project proposes to install a new low-energy storage ring in the ISAC-I experimental hall (see Fig. 8) and make use of stable beams from the offline laser ion source (OLIS) as well as brilliant, clean, and intense neutron-rich radioactive beams from the ISAC and ARIEL target stations.

The TRISR will be a storage ring of about 40–50 m circumference covering the energy range that can be injected from the ISAC-I acceleration chain ($\approx 0.15\,A$ MeV up to $1.8\,A$ MeV) for $A/q\le 7$. Higher energies would be accessible in the storage ring by re-acceleration with radiofrequency (RF) cavities and for lower *A*/*q*.

The momentum acceptance of the ring and the amount of ions that can be injected and stored are crucial parameters and need to be maximized. However, at the same time the injection of contaminants with similar *A*/*q* that could create an unwanted background in the neutron capture reactions needs to be avoided. For comparison, the TSR had a momentum acceptance of $\pm 3\%$ whereas the ESR has only $\pm 1.5\%$. We plan to store at least $10^{8}$ ions in the ring for the measurements.

The low beam energies down to $0.15\,A$ MeV require excellent UHV conditions ($p\ll 10 ^{-10}$ mbar) to allow a survival time of the orbiting beams in the order of seconds. The losses of highly-charged ions primarily occur due to interactions with residual gas in the beam line as well as with electrons in the electron cooler. For comparison, measured and calculated values of the TSR can be found in Table 15 in Ref. [61]: At $p=10^{-11}$ mbar, a cooled $^{80}\hbox {Se}^{25+}$ beam of $6\,A$ MeV could be stored for 204 s. A further advantage of a neutron target compared to a hydrogen or helium gas jet target is that the impact on the lifetime of the circulating beam is negligible, allowing for longer measurement times at lower energies.

Apart from UHV or even XHV conditions, phase-space cooling is a key technology for low energy storage rings to produce well-defined beam bunches in the ring with low momentum spread and small beam size. This can be achieved by beam cooling techniques like stochastic, laser, and electron cooling. While stochastic cooling is best suited for relativistic beams with $\beta >0.7c$, electron cooling is the method of choice for lower energies like in our TRISR. For a recent overview of these techniques, see Ref. [65].

The energy of the neutron capture reaction in inverse kinematics is controlled with the circulating beam energy. With electron cooling, the momentum spread $\delta p/p$ of the beam can be reduced by overlaying it with a “cold” electron beam. The process works like a heat exchanger and can achieve within a very short time (sub-seconds up to a few seconds, depending on the beam energy and initial energy spread) a momentum spread of $\delta p/p<10^{-3}$. The limiting factor is the duration for the cooling processes, especially for the use of short-lived radioactive beams. Electron cooling is very effective in reducing the momentum spread and can take several seconds for a radioactive beam originating from an in-flight fission and fragmentation reaction. For our ISOL beam that is charge-bred in the Electron Beam Ion Source (EBIS) this cooling time will be in the sub-second region. In addition, the electron cooler allows for small energy manipulations by adjustments of the cooler energy. In this way a scan over small energy ranges is possible without major re-tuning of the whole facility.

### The potential neutron target

As discussed for the LANL storage ring proposal [63], a spallation neutron source would be a viable but also very expensive solution as neutron target. For the TRISR project as shown in Fig. 8 this would require the construction of a new proton beamline from the main cyclotron as well as a building with heavy shielding for the spallation neutron source.

Thus a different approach is investigated with a high-flux neutron generator which will provide the moderated neutron “gas” target which is intersected by the circulating beam. While the intensity of the circulating beam is monitored with non-destructive Schottky pickups, the reacted beam will be detected in different ways, depending on the change of mass-to-charge ratio.

Various reaction-based compact neutron generator designs are presently discussed, among them the https://www.shinefusion.com/neutron-generators/d-t-deuterium-tritium-neutron-generators/ Alectryon 300T from SHINE Technologies, LLC which is presently the world’s strongest compact D-T neutron generator (producing up to $5\times 10^{13}$ n/s), with a gaseous target to maximize neutron yield and system lifetime. A dedicated design and feasibility study will be carried out in the next years to investigate this alternative solution.

### Reaction product detection

The sensitive detection of the neutron capture products poses another challenge. Charged-particle reactions in storage rings, e.g. as done for proton captures in the ESR [59, 60], allow the separation of the beam from the reaction products via the mass-to-charge difference by dipole magnets and subsequent detection with particle detectors. The change of the atomic number *Z* in these cases allows for a magnetic separation since the electron cooler reduces the momentum (velocity) spread of the ions in the ring.

This method is no longer possible for neutron capture reactions $^{A}$Z + $^{1}$n $\rightarrow$ $^{A+1}$Z where only the mass changes but not *Z*. While the mass difference could still be detected with Schottky detectors if the signal-to-noise ratio is high enough, a new method is being developed to extract the reaction products with a Wien filter followed by detection in a highly-sensitive recoil separator.

#### Schottky pickups

RF cavities are used since decades for non-destructive particle detection in storage rings. They are constructed as longitudinal cavities and can provide important time-resolved information on e.g. the beam revolution frequency, intensity changes, orbit shifts, and the extend of beam cooling processes.

In the ESR at GSI Darmstadt and other storage rings they are also used for mass measurements of the revolving ions via “Schottky mass spectrometry” and can also be used for the determination of decay half-lives [66]. In the ESR, a 245 MHz [67] and a 410 MHz [68] resonant Schottky cavity pickup have been installed and achieved single-ion sensitivity.

The combination of Schottky pickups for the monitoring of the circulating beam and particle detectors for the counting of the reaction products has been successfully applied for proton-capture reactions in Refs. [59, 60]. But for the operation at low energies in the TRISR the signal-to-noise ratio of the existing Schottky detectors is not (yet) sufficient. For the envisioned neutron capture reaction program a device is needed that is able to discriminate 1 reaction product ion against $10^{8}$ circulating unreacted ions.

The sensitivity of the existing resonant Schottky pickups [67, 68] for energies below $500\,A$ keV is unfortunately still very low. The GSI Storage Ring Group is presently designing new Schottky pickups (between the resonant and non-resonant regime) with lower bandwidth for operation in the CRYRING at energies similar to the TRISR. The TRISR project will benefit from this extensive R &D in the coming years.

#### Particle detectors

Reaction products that can be separated from the circulating beam by electromagnetic separation in dipole magnets can be intercepted and detected with high efficiency by particle detectors.

For example, the proton-capture experiments in the ESR [60] use a $16\times 16$-fold segmented, $500\,\mu \hbox {m}$ thick double-sided silicon strip detector (DSSD). Since the TRISR will work only at low energies (below 2 *A* MeV), a similar single-layer DSSD will be sufficient for stopping and detecting the reaction products. These (position-sensitive) DSSDs will be located at various locations inside the ring lattice behind the dipole magnets. They will be installed on a movable structure that allows the exact positioning with respect to the orbiting beam, and can be retracted into a safe parking position while not in use.

#### Recoil product extraction

A new method to extract the reaction products from the storage ring will be investigated here with the inclusion of a recoil separator into the storage ring matrix (see Fig. 8).

Since the recoil ions, immediately after the reaction, have the same average linear momentum as the beam but different mass (A+1), they have to be separated by *velocity*. In order to do this crossed perpendicular electric and magnetic fields, i.e. a Wien Filter, is required. The Wien filter causes true recoils to deviate from the beam trajectories by a relative angle that is dependent on the beam energy, beam mass and charge state. The unreacted beam particles pass straight through the Wien filter to continue their circulation in the ring while the recoils are separated from them.

The reaction products can be separated from the circulating beam via a Wien velocity filter followed by an extraction septum (e.g. Lambertson septum [69]). It should be emphasized that such a setup—the combination of a storage ring and a recoil separator—is a world-wide unique setup and will require a dedicated R &D feasibility study.

The philosophy is to design a method to continuously extract recoil ions from the circulating stored beam at a point immediately behind the neutron target. In this way, reaction products will be removed from the ring within a very short time after the radiative capture reaction takes place (on the order of $\mu \hbox {s}$) and transported to a secondary detection area using standard particle identification techniques such as $\varDelta \hbox {E}$–E, local time of flight, etc.

This method mimics the technique of proton-radiative-capture reactions using a recoil separator (such as the DRAGON facility at TRIUMF [70]) where recoil products are separated electromagnetically from the beam after impinging on a windowless gas target. It has the advantage that the efficiency of recoil detection does not depend on the recoil lifetime, which could be very short and affected by subsequent repetitive orbits in the ring. The method proposed here also has the advantage that little to no energy loss or scattering occurs on the neutrons in the target, leading to less scattered or ‘leaky’ beam that could be transmitted through the recoil separation device.

The separator must accept recoils up to 20 milliradians of half angle relative to the optic axis—a similar design acceptance to DRAGON [70]. This design will ensure that a very large range of possible ($n,\gamma$) reactions can be studied.

### Accessible isotopes

Not all isotopes in the chart of nuclei can be measured with the storage ring method due to different limitations. The most obvious limitation is of course the detection rate which depends on the number of ions that can be stored in the ring $\hbox {N}_\textrm{ring}$, the orbiting frequency (repetition rate) in the ring *f*(*E*), the neutron capture cross section $\sigma _{n,\gamma }(E)$, and the achievable neutron flux $\phi _n$ in the interaction zone:

1 $$\begin{aligned} N \propto N_\textrm{ring} \cdot f(E) \cdot \sigma _{n,\gamma }(E) \cdot \phi _n. \end{aligned}$$

The orbiting frequency *f*(*E*) and the neutron capture cross section $\sigma _{n,\gamma }(E)$ are energy-dependent. While the orbiting frequency increases with energy, e.g. it would be for the TRISR at 150 *A* MeV in the order of 100 kHz, the cross section decreases.

The ISOL method restricts access to elements and isotopes that can be (chemically) extracted within few ms from the target material. The shortest-lived isotope extracted so far at ISAC is $^{214m}\hbox {Fr}$ ($t_{1/2}=3.35$ ms). Elements with a high melting point, e.g. transition metals between Zr and Pd ($Z=40-46$) and Hf and Au ($Z=72-79$), or elements like B, P, Si, S cannot be extracted from ISOL targets in high quantities. Isotopes of these elements can thus not be studied at our facility.

Assuming an average measuring time of 10 s in the ring, we will thus focus on isotopes with a half-life of $t_{1/2}>5$ s which can be produced with a yield of at least $10^{7}$ pps. At the ISAC facility, more than 300 isotopes will be accessible in this range, including some long-lived isomeric states.

Examples for neutron capture measurements of astrophysical interest that could become accessible for a direct measurement with the TRISR are:

- *s*-process: $$^{134}\hbox {Cs}$$ ($$t_{1/2}=2.06$$ y), $$^{147}\hbox {Nd}$$ ($$t_{1/2}=11$$ d), $$^{148}\hbox {Pm}$$ ($$t_{1/2}=5.37$$ d), $$^{153}\hbox {Gd}$$ ($$t_{1/2}=240.4$$ d), $$^{160}\hbox {Tb}$$ ($$t_{1/2}=72.3$$ d), $$^{170}\hbox {Tm}$$ ($$t_{1/2}=128.6$$ d), and $$^{204}\hbox {Tl}$$ ($$t_{1/2}=3.78$$ y).
- *i*-process: $^{66}\hbox {Ni}$ ($t_{1/2}=54.6$ h), $^{75}\hbox {Ga}$ ($t_{1/2}=126$ s), $^{135}\hbox {I}$ ($t_{1/2}=$6.58 h), $^{141}$Ba ($t_{1/2}=18.27$ m), and $^{141}\hbox {La}$ ($t_{1/2}=3.92$ h).
- *r*-process: Nuclei at the neutron shell closures $N=50$ and 82, e.g. doubly-magic $^{132}\hbox {Sn}$ ($t_{1/2}= 39.7$ s), as well as neutron-rich nuclei in the rare earth peak region around $A\approx 160$.

The “true” range of accessible isotopes for the direct measurement of the neutron capture cross section in our TRISR will strongly depend on the achievable neutron densities in the interaction area. This can only be determined with a dedicated feasibility study of all components which will be initiated in 2023.

## Summary

The TRISR project proposes to design a world-wide unique facility that would allow for measuring neutron capture cross sections of short-lived radionuclides for the first time directly in inverse kinematics with a storage ring (TRISR) coupled to the existing TRIUMF-ISAC radioactive beam facility.

A high-intensity neutron generator in the ring matrix is suggested to create a quasi-static “neutron target” that intersects with the orbiting ion beam. This is a completely new concept and—if feasible—would create a pioneering facility for the measurement of neutron capture cross sections of radioactive nuclides and lead to a reduction of uncertainties from the neutron capture cross section in astrophysical models for the heavy element nucleosynthesis.

## Data Availability

This manuscript has no associated data or the data will not be deposited. [Authors’ comment:...].
